# A review on recent advances in the environmental occurrence of benzothiazoles: analytical methods and their role as non-exhaust traffic tracers

**DOI:** 10.1007/s11356-026-37952-0

**Published:** 2026-07-03

**Authors:** Matteo Feltracco, Elena Barbaro, Giovanna Mazzi, Andrei Munteanu, Mara Bortolini, Eleonora Favaro, Beatrice Ulgelmo, Elisa Scalabrin, Stefano Frassati, Carlo Barbante, Andrea Gambaro

**Affiliations:** 1 Department for Sustainability, Italian National Agency for New Technologies, Energy and Sustainable Economic Development (ENEA), Via Anguillarese, 301–00123 Rome, Italy; 2 Department of Environmental Sciences, Informatics and Statistics, Ca’ Foscari University of Venice, Via Torino, 155–30172 Venice Mestre, Italy; 3 Institute of Polar Sciences, National Research Council (CNR-ISP), Via Torino, 155–30172 Venice Mestre, Italy; 4 Department of Geosciences, University of Padova, Via Gradenigo, 6–35131 Padua, Italy

**Keywords:** Contaminants of emerging concern, Benzothiazoles, Rubber, Non-exhaust traffic emission

## Abstract

The study of benzothiazoles (BTHs) in the atmosphere is gaining attention due to their strong link with non-exhaust traffic emissions, particularly those resulting from tire wear. While these compounds have been widely investigated in water and soil, their presence in aerosol remains less explored, with reported atmospheric concentrations ranging from approximately 50 pg m^−3^ in remote areas up to 2000 pg m^−3^ in urban environments. Given their consistent detection in urban environments, BTHs could serve as effective markers for tracking non-exhaust traffic emissions, thereby enhancing our understanding of their contribution to air pollution and facilitating source identification. This review focuses on recent advances in the study of BTHs, providing an updated overview of their environmental occurrence and potential as traffic-related tracers. Additionally, it highlights the latest developments in analytical methods for their detection, emphasizing progress made in the past few years via UHPLC-MS/MS techniques, which achieve limits of detection down to sub-ng L^−1^ levels in aqueous matrices. This review concludes that to reliably employ BTHs as tracers, it is essential to analyse a broader range of derivatives and transformation products; such a comprehensive analytical approach is crucial for distinguishing between diverse emission sources and accurately accounting for the compounds’ environmental fate.

## Introduction

Benzothiazoles (BTHs) are used in diverse industrial applications, including as vulcanization accelerators in rubber production, biocides in paper and leather manufacturing, corrosion inhibitors in antifreeze formulations, and photosensitizers in photography (Kloepfer et al. 2005; Matamoros et al. 2010; Ni et al. 2008). Some pharmacological actions are shown by BTHs, as they act as inhibitors of biological targets (Chander Sharma et al. 2020). The first documented use of BTHs dates back to 1888 and it was published by Möhlau and Krohn (Möhlau and Krohn 1888). In this study, the authors explored the transformations of dimethylaniline and monomethylaniline under the influence of sulfur, leading to the synthesis of compounds containing the benzothiazole nucleus. Decades later, BTHs emerged as a critical component in industrial chemistry, particularly as accelerators in the vulcanization of rubber. Two studies by R. H. Campbell in 1964 (Campbell and Wise 1964a, 1964b) examined how BTHs significantly improved the efficiency of vulcanization by promoting faster cross-linking reactions. BTHs have thus garnered significant attention in recent decades, from both an industrial and an environmental point of view. They have been found in tire manufacturing plant wastewaters, urban stormwater runoff, estuarine sediments, and suspended river particles (Brownlee et al. 1992; Jungclaus et al. 1976; MacKenzie and Hunter 1979; Spies et al. 1987).

Subsequent studies have confirmed the widespread presence of BTHs in surface runoff sources and receiving rivers (Feltracco et al. 2023; Fries 2011; Kloepfer et al. 2005; Ni et al. 2008; Salas et al. 2016; H.-Y. Zhang et al. 2023a, b). Several BTHs are classified as toxic to aquatic organisms, with potential long-term adverse effects (Pillard et al. 2001; Tan et al. 2026). These compounds are highly water soluble, resistant to biodegradation, and only partially removed during wastewater treatment processes. The majority of studies in the literature highlight that wastewater treatment plants (WWTPs) exhibit only a minimal capacity to remove BTHs (Asimakopoulos et al. 2013a; Li and Kannan 2024; Matamoros et al. 2010; Reemtsma et al. 2006; Stasinakis et al. 2013; Wang et al. 2023; Xue et al. 2024). This limited removal efficiency raises significant concerns regarding the release of BTHs into the environment. Mass balance analysis and distribution coefficients reveal that some of the BTHs can undergo biotransformation during wastewater treatment (De Wever et al. 2001). Despite this, sorption onto biosolids and accumulation in sludge appear to play a negligible role in their removal (Asimakopoulos et al. 2013a). BTHs have been poorly studied in outdoor particulate matter samples, which may represent another significant pathway for human exposure (Barbaro et al. 2024). Liao et al. (Liao et al. 2018) report a table listing the main chemical and physical properties of some BTHs. Nevertheless, recent studies show that other BTHs may be released from tire wear particles (Kuntz et al. 2024). New studied BTHs (Akrochem 2023; Grung et al. 2021; Wu et al. 2024) are reported in Table 1. The list of BTHs provided could be further expanded but many transformation products, particularly those formed in the atmosphere, are still not well characterized (Karimova et al. 2024).

**Table 1:**
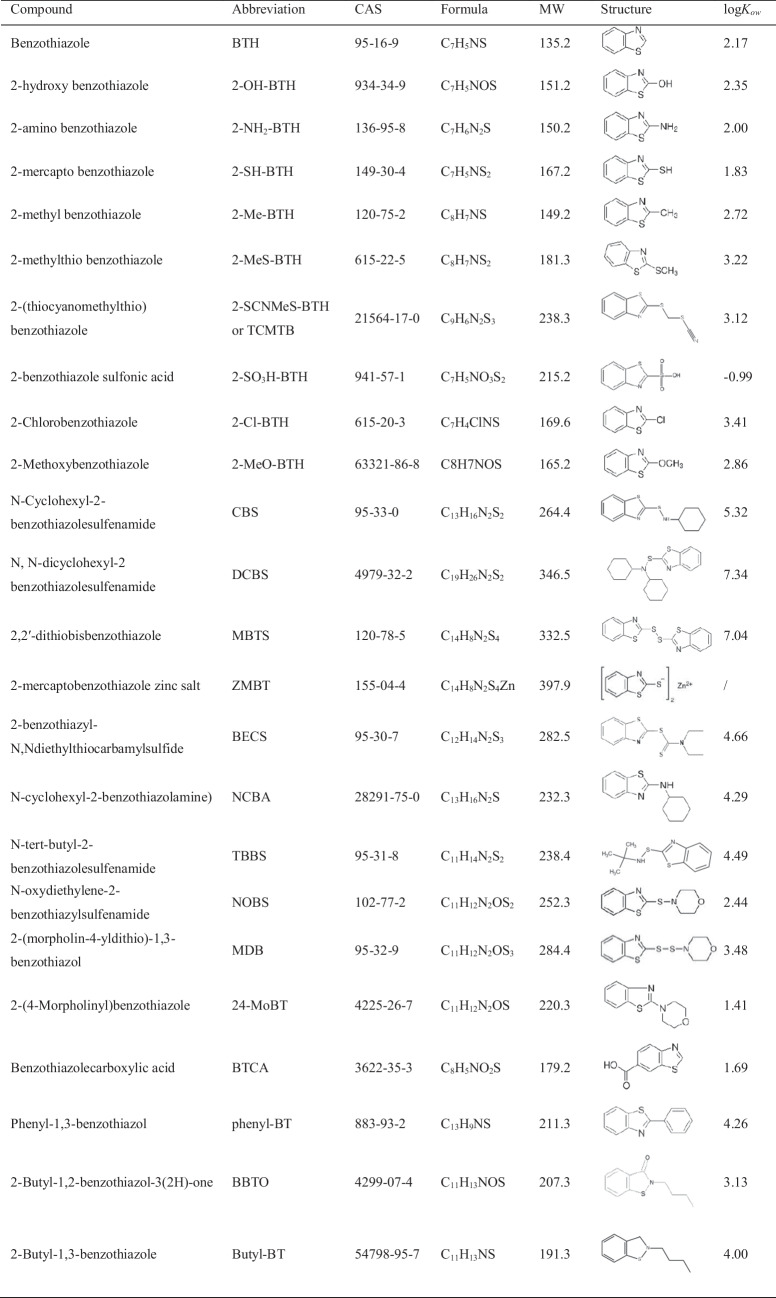
Comprehensive list of BTHs and relative information. *logK*_*ow*_ refers to the predicted data cited from chemspider.com

The environmental study of BTHs is a critical pillar in the global transition towards sustainable and resilient societies. Linking these findings to broader socioeconomic systems and resource governance is crucial. For instance, while electric vehicles eliminate tailpipe pollutants, their increased weight can lead to higher tire wear emissions compared to conventional vehicles, significantly affecting the overall monetary impact of the transition (Liu et al. 2022). Accurate monitoring is therefore a prerequisite for effective policy-making and the long-term viability of sustainable economic activities.

This review aims to provide an updated perspective on the role and the relevance of BTHs as tracers of non-exhaust vehicle emissions in different environmental matrices, considering the literature on this topic in the last 25 years. Specifically, it highlights the critical environmental challenges associated with BTHs in urban areas, primarily originating from tire wear. Thus, this work advances the field by: (i) focusing specifically on recent findings within aerosol matrices; (ii) synthesizing analytical developments from the last years and (iii) providing a critical evaluation of the tracer concept, addressing stability challenges such as leaching, volatilization, and the impact of non-traffic sources on tracer specificity. However, significant knowledge gaps remain regarding the detailed sources and health impacts of these compounds. Addressing these gaps is essential for developing effective mitigation strategies that ensure public health and environmental integrity.

## Analytical methodologies for BTHs determination

### Overview

**Fig. 1:**
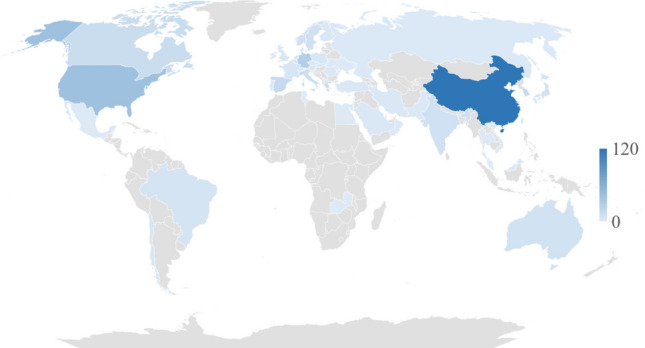
Compilation of studies published on BTHs analysis in various matrices per country since the year 2000. The used Scopus query is: *(TITLE-ABS-KEY (benzothiazole) AND TITLE-ABS-KEY (biota) OR TITLE-ABS-KEY (sediment) OR TITLE-ABS-KEY (urine) OR TITLE-ABS-KEY (serum) OR TITLE-ABS-KEY (blood) OR TITLE-ABS-KEY (human AND tissue) OR TITLE-ABS-KEY (soil) OR TITLE-ABS-KEY (leachate) OR TITLE-ABS-KEY (reservoir) OR TITLE-ABS-KEY (ground AND water) OR TITLE-ABS-KEY (sewage) OR TITLE-ABS-KEY (wastewater) OR TITLE-ABS-KEY (drinking AND water) OR TITLE-ABS-KEY (bottled AND water) OR TITLE-ABS-KEY (seawater) OR TITLE-ABS-KEY (river AND water) OR TITLE-ABS-KEY (lake AND water) OR TITLE-ABS-KEY (aerosol) OR TITLE-ABS-KEY (indoor AND aerosol) OR TITLE-ABS-KEY (dust) OR TITLE-ABS-KEY (suspended AND particulate AND matter) OR TITLE-ABS-KEY (snow) OR TITLE-ABS-KEY (ice)) AND PUBYEAR* > *1999 AND PUBYEAR* < *2026 AND (LIMIT-TO (SUBJAREA, "ENVI"))*

The earliest indications of BTHs occurrence in the environment date back to the 1970 s, with one of the pioneering studies being the work by Jungclaus et al. (1976). This research marked the first recognition of BTHs as environmental contaminants, providing insights into their detection and analytical quantification. These early findings laid the foundation for subsequent investigations into their environmental presence, sources, and potential impacts. Other articles have been published year after year in the 80 s and 90 s (Brownlee et al. 1992; Spies et al. 1987). The interest in BTHs significantly grew in the following decades, particularly in the context of wastewaters, as researchers began to focus on their behavior in aquatic environments and their removal rates in WWTPs. Studies expanded to include a broader range of BTHs compounds, investigating not only their persistence but also their transformation during treatment processes (Liao et al. 2018). This increasing focus highlighted the complexity of BTHs as a class of contaminants, emphasizing the need to better understand their fate, removal efficiency, and the formation of transformation products in the environment, which remain critical areas of ongoing research. A comprehensive bibliometric analysis was conducted using Scopus. The query, designed to capture studies on BTHs across various environmental and biological compartments, generated the map shown in Fig. 1. This analysis yielded 335 results, encompassing articles published from year 2000 onwards. The used query is reported below.

**Fig. 2:**
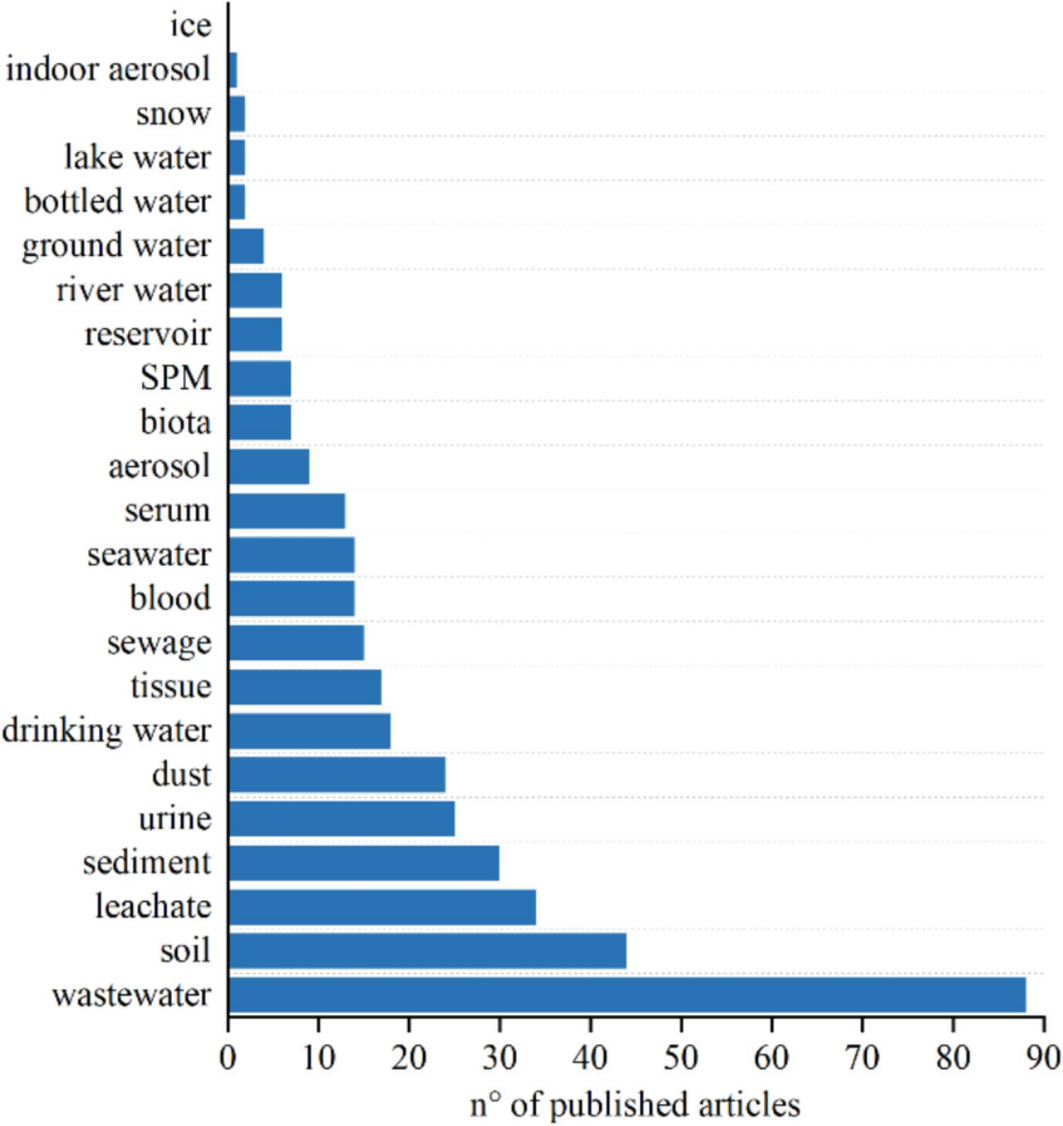
Studies published on BTHs analysis per matrix. SPM, suspended particulate matter

Furthermore, articles have been divided depending on the matrix type based on Scopus query results (Fig. 2). The majority of the studies retrieved focus on the determination of BTHs in wastewater (Kilpinen et al. 2023; Li and Kannan 2024; Ng et al. 2023; Xue et al. 2024; H.-Y. Zhang et al. 2023a, b) or soil leachate and sediment (Kim et al. 2022b; Li et al. 2023; Thomas et al. 2022; Wik and Dave 2009). This emphasis is largely due to the extensive research on their release in urban environments, where the primary source of BTHs is linked to tire wear particles. This urban-centric focus in literature underscores the importance of understanding the pathways and impacts of BTHs stemming from this dominant source. In general, the BTHs most analyzed in the environment are BTH, 2-OH-BTH, 2-Me-BTH, 2-NH_2_-BTH and 2-SCNMeS-BTH, which are also the most used BTHs industry wide.

### Water

Water samples are very often pretreated by adjusting to low pH with acids and by spiking with Na_2_EDTA. They are then passed through SPE cartridges (i.e., Oasis HLB, Strata-X, Bond Elut-PPL), with different solvents (Asheim et al. 2019; de Mendonça Ochs et al. 2023; Xue et al. 2024; R. Zhang et al. 2023a, b). These procedures are commonly applied also for seawater (Gutiérrez-Martín et al. 2023; Zhao et al. 2024b, 2024a). Modifications to the classic SPE system have also been tested, including the use of a multilayer solid-phase extraction (ml-SPE) approach with different sorbents, specifically, Oasis WAX, Oasis MCX and Isolute ENV + cartridges (Kilpinen et al. 2023). Stir bar sorptive extraction (SBSE) as preconcentration was also applied in a previous study, followed by a thermal desorption and GC/MS analysis (Mol et al. 2024). Thus, most of the determinations were performed by UHPLC-MS/MS. Additionally, liquid–liquid extraction and large-volume solid-phase extraction (LVSPE) sampling system has been applied to allow the processing of larger sample volumes, thereby lowering the detection limits of the analysis (Maurer et al. 2023). The process was further simplified through direct injection of stormwater, prior to dilution (Feltracco et al. 2023). It is evident that applying direct injection, or even sample dilution, requires careful consideration to ensure the detection of the target analytes. Matrix effects should be also carefully investigated.

The inconsistency among studies, which often did not analyze the same set of BTHs, makes it challenging to compare concentrations across different environmental compartments. Generally, BTH is frequently detected at concentrations as high as 20,000 ng L^−1^ in urban wastewater (Xue et al. 2024; H.-Y. Zhang et al. 2023a, b). In contrast, its concentrations in Chinese seawater are significantly lower, reaching up to only 6 ng L^−1^ (Zhao et al. 2024a). The wide range of concentrations observed, spanning several orders of magnitude, reflects the complex interplay of urbanization intensity, proximity to point sources, and local hydrological conditions. This disparity highlights the variability in environmental distribution and emphasizes the need for more comprehensive and standardized approaches in BTHs analysis.

### Soil

As shown in Fig. 2, research on BTHs in soil has become increasingly prevalent, especially in recent years; however, these studies remain significantly fewer compared to those focusing on the dissolved fraction. Soil and road dust are an important sink of tire wear particles and play a significant role in their transportation and degradation in the environment. Several methods have been proposed to quantify the BTHs content therein. After collection, soil samples are passed through a mesh sieve in order to discriminate the samples depending on the particle diameter (Feltracco et al. 2023; Zhang et al. 2018) or to homogenize the samples to achieve higher reproducibility (Núñez et al. 2022). Cryogenic milling can also be employed to prepare particulate samples for analysis, aiming to the production of particles with appropriate dimensions for subsequent chemical analyses, enhancing the accuracy and reliability of the obtained results. After the sieving process, even in the most recent studies, BTHs are typically extracted using sonication with ultrapure water (Feltracco et al. 2023), water/methanol mixtures (Zhang et al. 2018), 100% methanol (Deng et al. 2022) or 100% 2-propanol (Klöckner et al. 2020). At this stage, many studies proceed directly with injection for analysis, while others include a purification step using SPE (Zhang et al. 2018) to enhance sample quality. Accelerated Solvent Extraction (ASE) is also used as a method for extracting organic phase, using high temperature and pressure to efficiently isolate compounds. Asheim et al. (2019) (Asheim et al. 2019) outlined an extraction method for BTHs from bitumen and tire samples, involving dissolution in DCM, addition of an acidified water/methanol solution, and sonication. As with water, the vast majority of studies use UHPLC-MS/MS systems for BTHs determination (Table 2). The shift from GC to LC in BTHs analysis is primarily driven by the physicochemical properties of these analytes. Most BTHs and their transformation products are highly polar and exhibit low volatility. GC analyses require a mandatory derivatization step, increasing the complexity and duration of sample preparation and introducing artifacts or low recovery rates. Concentrations of BTHs can vary widely, ranging from a few tens of ng g⁻^1^ in road dust (Feltracco et al. 2023) to as high as 2900 ng g⁻^1^ in roadside samples, while samples collected elsewhere typically exhibit concentrations that are orders of magnitude lower.

**Table 2:** Recent published papers on BTHs determination in environmental matrices and their respective techniques and methods. LOD and LOQ concentrations are reported in ng L^−1^ for liquid samples, ng g^−1^ for solid samples and pg m^−3^ for aerosol. The literature search covered the period from January 2010 to mid-2026 following the PRISMA guidelines transparency principles. The search query focused on BTHs combined with specific environmental matrices. Duplicate records were automatically removed

Matrix	BTHs	Location/sample type	Sample preparation	Instrumentation	Column	LOD	LOQ	Ref
Waste, river and seawater	BTH, 2-NH_2_-BTH, 2-Me-BTH, 2-MeS-BTH, 2-SCNMeS-BTH, NCBA	Liuxi River, China	SPE	UHPLC-MS/MS	Waters XBridge BEH C18	0.007–3.60	0.02–10.8	(R. Zhang et al. 2023a, b)
	BTH, 2-OH-BTH, 2-MeS-BTH	Western South China sea	SPE	GC–MS	Agilent DB-5MS	0.03–0.21	0.12–0.69	(Zhao et al. 2024b, 2024a, 2024c)
	BTH, 2-OH-BTH, 2-MeS-BTH	Pearl River Estuary, China	SPE	UHPLC-MS/MS	Waters BEH C18	0.83–8.30	2.00–28.00	(Wei et al. 2024)
	BTH, 2-SH-BTH, MBTS, CBS, DCBS, NOBS, TBBS	Pearl River, China	SPE	UHPLC-MS/MS	/	0.01–2.69	0.02–8.95	(H.-Y. Zhang et al. 2023a, b)
	BTH, 2-NH_2_-BTH, 2-SH-BTH	Jacarepaguá Lagoon, Brazil	SPE	UHPLC-MS/MS	Waters Acquity BEH C18	0.4–3.0	1.2–10	(de Mendonça Ochs et al. 2023)
	BTH	Danish wastewater	ml-SPE	GCxGC-QTOF	Phenomenex non-polar ZB-5 + mid-polar ZB-50	0.3	0.5	(Kilpinen et al. 2023)
	BTH, 2-OH-BTH, 2-NH_2_-BTH, 2-MeS-BTH, NOBS	WWTP, New Yok, USA	SPE	UHPLC-MS/MS	Restek Ultra AQ C18	0.05–10	/	(Li and Kannan 2024)
	BTH, 2-MeS-BTH, 2-SO_3_H-BTH, 2-SCNMeS-BTH, 2-OH-BTH, 24-MoBT, 2-Me-BTH, NCBA	Aachen, Germany	Direct injection	UHPLC-OrbitrapMS	Phenomenex Kinetex EVO C18	/	/	(Fuchte et al. 2022)
	BTH, 2-NH_2_-BTH, 2-Me-BTH, 2-MeS-BTH, 2-SH-BTH, 2-OH-BTH, 2-SCNMeS-BTH, 2-SO_3_H-BTH	Venice, Italy	Direct injection	UHPLC-MS/MS	Thermo Betasil C18	2.0–5.0	6.0–18.0	(Feltracco et al. 2023)
	BTH, 2-OH-BTH, 2-MeS-BTH, 2-SH-BTH, 2-NH_2_-BTH, 24-MoBT, 2-Cl-BTH, 2-SCNMeS-BTH, 2-Me-BTH	Glacier-fed river, Arctic	pH adjustment to pH < 3, filtration	UHPLC-MS/MS	Phenomenex Kinetex C18 column	0.33–5.0	1.0–15	(Amey and Mikkelsen 2025)
	2-OH-BTH, phenyl-BT, BBTO, CBS	Norway	SPE	UHPLC-QTOF	Waters BEH C8	0.3–0.33	0.1–1.0	(Grung et al. 2021)
Soil and sediment	BTH, 2-OH-BTH, 2-NH_2_-BTH, 2-SH-BTH, 2-MeS-BTH,	Simulated abrasion of tires	Sonication + SPE	UHPLC-MS/MS	Waters BEH C18	3.0–120	10–400	(Zhang et al. 2018)
	BTH, 2-NH_2_-BTH, 2-Me-BTH, 2-MeS-BTH, 2-SH-BTH, 2-OH-BTH, 2-SCNMeS-BTH, 2-SO_3_H-BTH	Venice, Italy	Direct injection	UHPLC-MS/MS	Thermo Betasil C18	0.012–1.562	0.42–5.21	(Feltracco et al. 2023)
	BTH, 2-OH-BTH, 2-MeS-BTH, MDB, 2-Cl-BTH	Road dust and parking, China	Sonication	UHPLC-MS/MS	Phenomenex Kinetex C18	0.13–67.2	0.4–120.0	(Deng et al. 2022)
	2-SH-BTH, NOBS, 2-MeS-BTH, 2-OH-BTH, 2-NH_2_-BTH	Road dust, Germany	Sonication	UHPLC-QTOFMS	Waters Acquity HSS T3	/	3.0–10.0	(Klöckner et al. 2020)
	BTH, 2-SH-BTH, 2-OH-BTH, 2-MeS-BTH, 2-Me-BTH, 2-NH_2_-BTH	Norwegian bitumen and tyres	DMC dissolution, sonication	UHPLC-MS/MS	Waters Atlantis C18 T3	1.5–16.5	5.0–50.0	(Asheim et al. 2019)
	BTH, 2-OH-BTH, 2-MeS-BTH, 2-SH-BTH, 2-NH_2_-BTH, 24-MoBT, 2-Cl-BTH, 2-SCNMeS-BTH, 2-Me-BTH	Marine and fluvial sediments, Arctic	Sonication	UHPLC-MS/MS	Phenomenex Kinetex C18 column	0.33–3.3	1.0–10	(Amey and Mikkelsen 2025)
	BTH, 2-OH-BTH, 2-NH_2_-BTH, 2-MeS-BTH, NOBS	Surface and core sediments, Lake Sihwa, Korea	Three-time sonication and shaking	UHPLC-MS/MS	Restek Ultra AQ C18 column	0.416–8.17	/	(Jeong et al. 2026)
	BTH, 2-OH-BTH, 2-MeS-BTH	Yangtze River Estuary, China	Twice shaking	GC–MS	Agilent DB-5MS	0.03–0.21	0.09–0.69	(Zhao et al. 2024c)
Leachate	BTH, Me-BTH, MeS-BTH, CBS, MTBS	Tyre abrasion	Double ultrasonic extraction	UHPLC-MS/MS	ACE C8	/	0.10–0.14	(Ren et al. 2024)
	BTH	Tyre abrasion	Shaking with lightproof vibrator	UHPLC-QTOF/MS	/	/	/	(Li et al. 2024)
	2-Cl-BTH, BTH	Tyre abrasion	Shaking with lightproof vibrator	GC-TOFMS	Agilent DB-5MS UI	/	/	(Kim et al. 2023)
Urine	BTH, 2-OH-BTH, 2-NH_2_-BTH	Japan, Korea, China	Enzymatic deconjugation + SPE	UHPLC-MS/MS	Waters X Terra MS C18	70.0–4000	200–5000	(Asimakopoulos et al. 2013b)
	BTH, 2-OH-BTH, 2-NH_2_-BTH, 2-MeS-BTH, 2-SCNMeS-BTH	Australia	Enzymatic deconjugation + centrifugation	UHPLC-MS/MS	Phenomenex Kinetex biphenyl	8.6–1200	30–4100	(Que et al. 2024)
Aerosol	BTH, phenyl-BT, 2-MeS-BTH,	California, USA	Headspace collected water through headspace	APCI-Orbitrap-MS/MS	/	/	/	(Franklin et al. 2021)
	BTH, 2-NH_2_-BTH, 2-OH-BTH, CBS, 24-MoBT, BTCA, 2-SO_3_H-BTH, 2-SH-BTH, NOBS	PM10, Germany	Sonication	UHPLC-MS/MS	Waters HSS T3	/	0.021–2328	(Kuntz et al. 2024)
	BTH, 2-NH_2_-BTH, 2-Me-BTH, 2-MeS-BTH, 2-SH-BTH, 2-OH-BTH, 2-SCNMeS-BTH, 2-SO_3_H-BTH	Size-segregated Indoor aerosol, Venice	Sonication	UHPLC-MS/MS	Thermo Betasil C18	0.04–0.60	0.03-	(Feltracco et al. 2024)
	BTH, 2-NH_2_-BTH, 2-Me-BTH, 2-MeS-BTH, 2-SH-BTH, 2-OH-BTH, 2-SCNMeS-BTH, 2-SO_3_H-BTH	Size segregated aerosol, Venice	Sonication	UHPLC-MS/MS	Thermo Betasil C18	0.04–0.60	0.03–1.99	(Favaro et al. 2024)
	BTH, 2-NH_2_-BTH, 2-Me-BTH, 2-MeS-BTH, 2-SH-BTH, 2-OH-BTH, 2-SCNMeS-BTH, 2-SO_3_H-BTH	PM10, Arctic	Sonication	UHPLC-MS/MS	Thermo Betasil C18	0.04–0.60	0.03–1.99	(Barbaro et al. 2024)

### Leachate

Leaching from tire rubber includes microplastics, phthalates, phenols and also BTHs, where the latter are typically observed in the highest quantities (Capolupo et al. 2020). While numerous chemical compounds can leach from rubber and plastic, they may be present at concentrations below analytical detection limits. However, BTHs, being established markers and often identified as predominant compounds in this context, can be readily used for such studies. To produce leachates for chemical characterization, samples were agitated under controlled conditions, typically in a liquid medium, at various temperatures and also in the absence of light (Halsband et al. 2020; Kim et al. 2023; Li et al. 2024). An interesting test was performed by Kim et al. (2022); (Kim et al. 2022a) that used sole fragments immersed in an aqueous solution and agitated under controlled temperature, speed, and dark conditions for 29 days to facilitate leachate generation. For GC analyses, leachate was then extracted with apolar solvents, while for HPLC it was commonly direct injected upon filtration. Due to the hydrophilicity of BTHs, their concentrations in leachate can reach levels as high as 37 µg L^−1^ (Ren et al. 2024).

### Other matrices

The extraction procedures for BTHs in sediments are very similar to those used for soils, typically involving ultrasonication with solvents such as methanol and acetonitrile, followed by purification using SPE. For urine analysis, samples are typically incubated with ammonium acetate buffer and β-glucuronidase/sulfatase and adjusted to low pH. After extraction on preconditioned SPE, target compounds are eluted with methanol or acetonitrile, concentrated under nitrogen, and reconstituted with water solution for instrumental analysis (Li et al. 2018; Mao et al. 2024). Li and Ding (2021) carried out the deep eutectic solvent with ultrasound-assisted liquid–liquid microextraction (DES-UALLME) procedure under optimal conditions after enzymatic hydrolysis of the urine samples. Specifically, DES and NaCl were added to the sample, which was adjusted to low pH. The sample was manually shaken to dissolve the NaCl before proceeding with UALLME. A key advantage of the procedure is its use of only 300 μL of DES, making it low-cost and environmentally friendly by avoiding the use and generation of hazardous substances. In the majority of studies in the literature that address the determination of BTHs in sediments, it has been observed that the high water solubility resulted in lower concentrations in this matrix, despite their detection at elevated levels in polluted aqueous phase (Feltracco et al. 2023; Xu et al. 2022; Zhao et al. 2024b, 2024a, 2024c). This is attributable to the tendency of BTHs to remain more concentrated in the water phase due to their solubility, thereby reducing the extent of their accumulation in the sediment phase. Among the BTHs, BTH itself is the most concentrated, with levels reaching up to 23,000 ng L^−1^ (Liao et al. 2018; Que et al. 2024).

### Selection criteria for analytical workflows

Choosing the optimal extraction method for BTHs requires balancing matrix complexity with the chemical properties of the target analytes. For aqueous matrices, SPE remains the gold standard due to its ability to process large sample volumes and provide high enrichment factors. Standard sorbents like Oasis HLB are preferred for their versatility in capturing both parent BTHs and polar derivatives. However, for high-concentration matrices like wastewater, direct injection coupled with mass spectrometry is emerging as a faster, solvent-free alternative that minimizes sample loss. For solid matrices such as soil, sediment, and road dust, the primary challenge is the release of analytes from the tire rubber or mineral particles. Ultrasonication with organic solvents (methanol or acetonitrile) is the most effective approach for ensuring high recovery rates. This step must be followed by SPE cleanup to reduce matrix effects, which are particularly severe in aerosol and road dust samples. In biological monitoring, while SPE is common, DES-UALLME offers an ideal method when small sample volumes (e.g., hundreds of µL) are available and high sustainability is required. In the future, the adoption of micro-SPE and online-SPE techniques should be prioritized to further reduce pre-analytical times and minimize sample and solvent consumption.

**Fig. 3:**
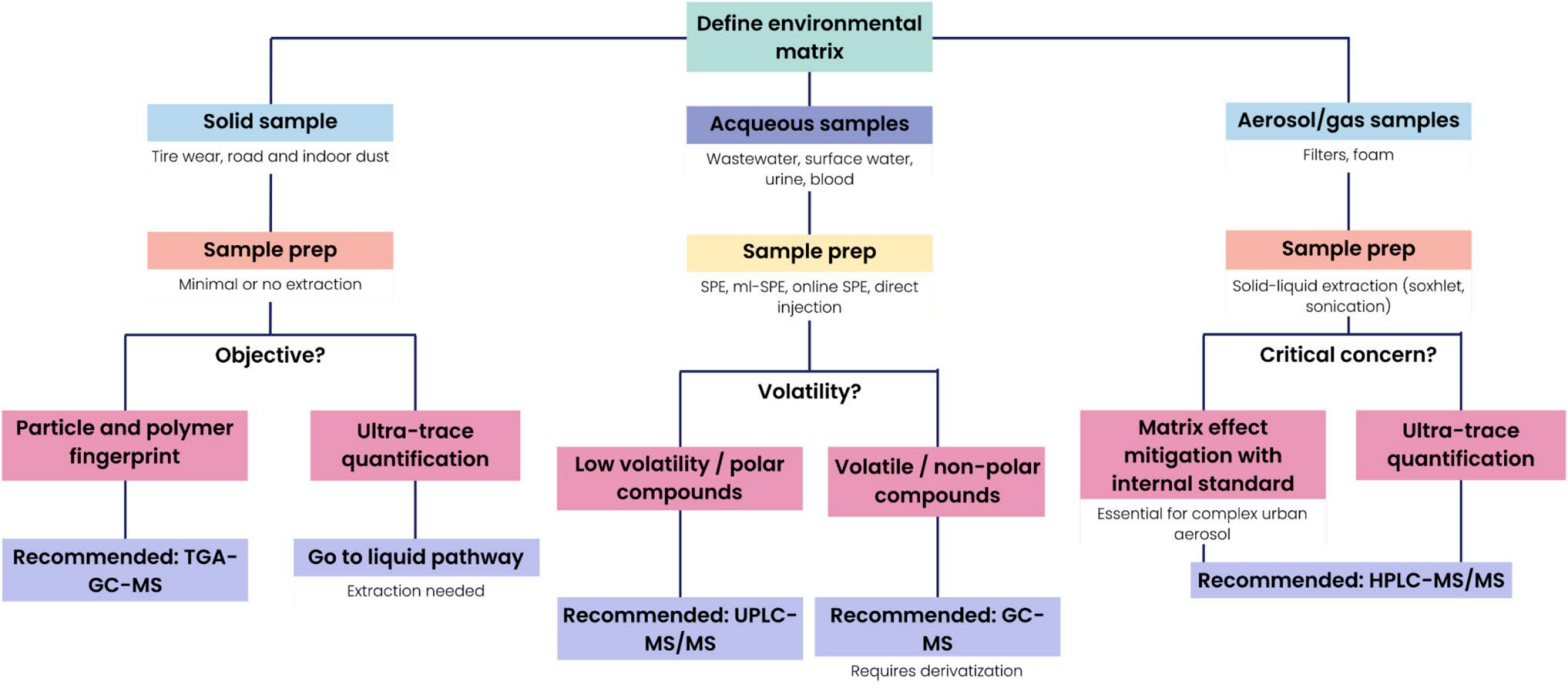
Decision tree for the selection of BTHs analytical methodologies based on environmental matrix characteristics and objectives

Method performance characteristics vary significantly across different environmental matrices. In complex aerosol samples, matrix effect is often more pronounced than in wastewater due to the high load of organic and inorganic interferences, which can systematically bias concentrations if not properly corrected through internal standards or matrix-matched calibration. However, a systematic cross-matrix comparison remains challenging as many studies fail to report detailed performance characteristics. This lack of transparency leads to high variability in LOD and LOQ values, which can differ by orders of magnitude between studies, complicating the comparison of BTHs concentrations across different environmental compartments. To guide researchers in selecting the most appropriate analytical pathway, we propose a decision framework based on the sample matrix and research objective. For solid rubber-related samples, TGA-GC/MS is recommended to bypass complex extraction steps. Conversely, for aqueous and aerosol samples where BTHs are present at trace levels, UHPLC-MS/MS remains the standard due to its ability to handle polar BTH derivatives without the need for derivatization. In complex matrices, the inclusion of isotopically labeled internal standards is mandatory to mitigate significant matrix effects. To synthesize these analytical considerations, a decision-making framework is proposed in Fig. 3.

## BTHs as traffic-derived non-exhaust tracers in aerosol

### Aerosol and toxicity

BTHs once released into the environment, lead to potential health risks through inhalation. The distinct volatility and propensity for aerosolization of benzothiazoles raise urgent concerns regarding indoor air quality, particularly in settings where these compounds are used or produced, such as in rubber manufacturing facilities or automotive industries (Liao et al. 2018). Recent studies have demonstrated that benzothiazoles, including key representatives like 2-SH-BTH and 2-OH-BTH, can be found in indoor air samples (Feltracco et al. 2024; Ginsberg et al. 2011). The inhalation of these aerosolized compounds poses health risks, including respiratory irritation and sensitization, which are exacerbated by their potential to induce oxidative stress and inflammation in lung tissue. For example, research has shown that exposure to 2-SH-BTH can lead to cellular and tissue remodeling processes linked with respiratory diseases, particularly in workers with chronic exposure. The underlying mechanisms involve inflammatory responses due to oxidative damage, activating pathways associated with cell death and chronic lung conditions (Liao et al. 2018). Epidemiological studies have further elucidated the carcinogenic potential of BTHs, particularly among populations exposed to them in occupational settings. For instance, investigations involving rubber factory workers and those in industries utilizing BTHs have identified a higher incidence of respiratory cancers, including lung carcinoma and bladder cancer. These findings emphasize the critical need for continuous air quality monitoring and intervention strategies to mitigate BTHs exposure, particularly in enclosed environments where aerosol concentrations can accumulate significantly (Ginsberg et al. 2011). Notably, the ecological ramifications of aerosolized BTHs cannot be overlooked. These compounds can deposit into terrestrial and aquatic environments, contributing to chemical loading in waterways through atmospheric deposition (Núñez et al. 2020; R. Zhang et al. 2023a, b). Studies using model organisms like zebrafish have illustrated how exposure to benzothiazoles can lead to developmental issues, such as impaired hatching and altered behavioral responses due to neurotoxicity. The regulatory frameworks governing benzothiazole usage must also adapt to address the hazards posed by aerosolized forms. Current regulations may not comprehensively account for the inhalation risks associated with benzothiazoles, particularly in industrial settings where emissions can lead to airborne contamination. Implementing stringent occupational exposure limits, coupled with improved ventilation and air quality standards in workplaces where benzothiazoles are used, is critical to protecting workers’ health (Yin et al. 2024). Furthermore, BTHs can serve as chemical proxies for the complex mixture associated with tire wear particles (TWPs). As unique markers of the vulcanization process, their presence indicates exposure to the broader “chemical cocktail” of TWPs, including metals and other toxic additives, facilitating a more comprehensive assessment of the synergistic health risks of non-exhaust emissions. However, a clear distinction must be made between occupational settings, where exposure levels can reach higher ranges than ambient environments. At present, the toxicological relevance of these lower ambient concentrations remains largely uncharacterized, making any definitive health risk assessment for the general population purely speculative.

The toxicity data compiled in this review highlights a critical regulatory gap, as traditional air quality standards focus almost exclusively on exhaust emissions. Regulation (EU) 2024/1257 represents a landmark shift by introducing the first mandatory limits for brake and tire abrasion. However, the specific chemical risks identified here suggest that mass-based limits alone are insufficient to address biological impacts. To bridge this, regulatory bodies must adopt standardized methodologies like the UN Global Technical Regulation (GTR) No. 24. Integrating these standardized markers into policy will allow for a transition from bulk mass metrics toward health-protective standards based on the specific toxicological profile of non-exhaust particles.

### Occurrence of BTHs

The presence and distribution of BTHs across various environmental compartments, particularly in wastewater, are well-documented to date. However, a critical challenge in the studies conducted across the matrices illustrated in Fig. 2 lies in the inconsistency regarding the number and types of BTHs analyzed. This lack of standardization poses significant difficulties in comparing relative concentrations among published studies, ultimately complicating their investigation and undermining the reliability of BTHs as markers for traffic-derived non-exhaust emissions. Non-exhaust emissions, such as tire wear particles and brake wear particles, are significant contributors to environmental pollution, particularly in urban areas. Unlike exhaust emissions, which are well-studied and regulated, non-exhaust emissions remain poorly characterized but are increasingly recognized for their impact (Fussell et al. 2022). Understanding the sources and distribution of these emissions is crucial for assessing their environmental and health risks, as they contribute to airborne and waterborne contaminants with persistent, widespread effects. Identifying specific markers for non-exhaust emissions, particularly in aerosol, is crucial for accurately quantifying traffic’s contribution to atmospheric pollution. These markers help distinguish non-exhaust pollutants, enabling precise assessments of their role in air quality and environmental health. BTHs can play a crucial role, as outlined in a few studies published recently. Kuntz et al. (2024) (Kuntz et al. 2024) determined nine BTHs in atmospheric aerosol of Germany, with a total median concentration of 204 pg m^−3^. 2-SO_3_H-BTH was the most abundant (61% of total), followed by 24-MoBT (20%), and BTH (11%). 2-SO_3_H-BTH and BTCA were detected for the first time in this matrix. Other studies have found them in both size-segregated outdoor and indoor aerosol in Italy (Favaro et al. 2024; Feltracco et al. 2024) and in the PM_10_ in the Arctic (Barbaro et al. 2024). Although the investigated BTHs in these studies were the same, results were different in terms of relative abundance. In an urban outdoor aerosol of Venice (Favaro et al. 2024) eight BTHs were determined, revealing their predominance in fine particles (< 1.8 µm) and highlighting their potential as traffic-related markers and contributors to atmospheric processes. 2-SH-BTH and BTH were the most concentrated compounds, with a mean value of 300 pg m^−3^. The same BTHs were also investigated in a size-segregated indoor aerosol of Venice, showing 2-SO_3_H-BTH and 2-SH-BTH as the most concentrated compounds with a mean value of 2000 pg m^−3^, predominantly present in fine particles (< 0.56 μm) (Feltracco et al. 2024). When using BTHs as tracers for tire wear particles and non-exhaust emissions, it is crucial to account for their leaching and volatilization processes, which significantly influence their atmospheric behavior. While tire wear particles are predominantly found in the coarse fraction, BTHs have been observed also in the fine fraction (< 0.56 µm), as reported in the study conducted in Venice. This discrepancy is likely due to the volatilization of BTHs during particle braking events, followed by their condensation and adsorption onto finer aerosol particles. These processes must be carefully considered to ensure the reliable use of BTHs as markers for tire wear particles and non-exhaust emissions. The key indoor sources included textiles, shoe abrasion, and building materials. In the Arctic aerosol (Barbaro et al. 2024), the most abundant compound was 2-OH-BTH, representing a mean concentration of about 50 pg m^−3^. The main reason for these differences is not completely investigated but a speculation can be different sources or photochemical transformation during the atmospheric transport processes (Liao et al. 2018).

To effectively use BTHs as non-exhaust traffic tracers, it is essential to analyze the full range of BTHs (as shown in Table 1). Relying on a limited subset may not guarantee that the identified source is solely traffic-derived, especially in aerosol where oxidation processes can occur rapidly, altering the chemical composition. Therefore, a comprehensive determination of all relevant BTHs is necessary to ensure accurate and reliable identification of traffic-related non-exhaust emissions. Distinguishing tire wear from other industrial sources using BTHs profiles is a complex task that has yet to be fully realized through standardized diagnostic ratios. However, recent evidence suggests that a multi-compound approach significantly enhances source attribution. For instance, in the Arctic environment, Barbaro et al. (2024) identified natural/biogenic sources, specifically linked to 2-OH-BTH and 2-NH_2_-BTH, and successfully separated them from anthropogenic signals and long-range transport (BTH and 2-SO_3_H-BTH). Similarly, research at Milan Linate airport (Mazzi et al. 2026) identified airport-specific signatures, linking 2-NH_2_-BTH and 2-SO_3_H-BTH to de/anti-icing procedures and aviation activities. Furthermore, in high-altitude alpine sites, the dominance of 2-SO_3_H-BTH was used to confirm tire wear resuspension as the primary source, effectively separating this anthropogenic signature from biogenic and crustal constituents (Favaro et al. 2026). While specific BTHs-to-BTHs ratios are not yet universal, these studies demonstrate that the presence and relative abundance of different BTHs derivatives, potentially in combination with inorganic markers like zinc from the vulcanization process, are key to refining source apportionment models.

Accurately tracing tire wear emissions through BTHs is fundamental for fair resource allocation and urban governance. Identifying pollution sources with precision is the cornerstone of the “polluter pays” principle, allowing for the design of equitable regulations and urban planning strategies, such as Low-Emission Zones (LEZs). Research in cities like Rome has demonstrated that such data-driven traffic policies can lead to measurable social welfare benefits, including significant gains in life expectancy across different socioeconomic groups (Cesaroni et al. 2012). By setting BTHs within this regulatory framework, their role as indicators shifts from a chemical metric to a tool for public health protection and social equity.

### Challenges and perspectives

A critical limitation in using BTHs as tracers for TWPs lies in their environmental fate and semi-volatile nature. As noted previously, BTHs can undergo volatilization from coarse TWPs followed by re-condensation or adsorption onto finer, pre-existing aerosols. This process can decouple the BTHs signal from the actual mass of the TWPs, particularly in the fine fraction. If BTHs originally associated with coarse particles re-condense on non-tire-related fine aerosol, the resulting signal may lead to an overestimation of the contribution of fine tire wear particles to total mass. Moreover, atmospheric transformations, such as the oxidation of BTH to 2-OH-BTH and 2-SO_3_H-BTH (Liao et al. 2018), can further alter the original tracer profile. These dynamic processes mean that BTHs concentrations in aerosol reflect a combination of direct emission and secondary redistribution, which must be considered when evaluating their fidelity as tracers for the particles themselves. Establishing a robust quantitative relationship between total BTHs concentrations and the actual mass of tire wear in the environment remains a significant challenge. Currently, BTHs are primarily used as qualitative markers rather than quantitative proxies. This is largely due to the high variability of BTHs concentrations across different tire brands and formulations.

Conventional methods such as Pyrolysis–Gas Chromatography-Mass Spectrometry (Pyr-GC/MS) directly target the rubber polymer backbone. While this approach provides a more direct measure of particle mass, it requires larger sample volumes and can suffer from interferences with other polymers, such as bitumen. Novel techniques like the thermogravimetric analysis TGA-GC/MS (Evans et al. 2025), offer faster analysis and the detection of specific additives like 6-PPD, which is highly specific but subject to rapid environmental degradation. In contrast, BTHs analyzed via UHPLC-MS/MS provide exceptional sensitivity, allowing for detection in low-volume or remote aerosol samples where polymer mass might fall below detection limits.

One of the most significant gaps concerns the atmospheric fate of BTHs. Recent studies have demonstrated that BTHs are not chemically inert; they undergo rapid photochemical oxidation initiated by hydroxyl radicals. H-abstraction is the dominant pathway for the atmospheric degradation of 2-OH-BTH, leading to a variety of ring-opening and substitution products (Jahanzab et al. 2025). Furthermore, the aqueous-phase chemistry in clouds and fogs, driven by oxidation and oligomerization mechanisms, represents an additional, understudied source of secondary aerosol mass (Zhan et al. 2025).

There is a profound lack of studies investigating the population-level health risks associated with chronic, low-level inhalation of BTHs in urban aerosol. While the presence of BTHs in PM is well-documented, the long-term respiratory and systemic effects of inhaling these vulcanization accelerators remain unknown. Future work should prioritize epidemiological studies and in vitro lung models to establish safe ambient exposure limits. Furthermore, the detection of BTHs in remote regions like the Arctic (Barbaro et al. 2024) underscores their potential as markers for the long-range atmospheric transport of urban pollutants. This discovery opens a new research frontier: using BTHs as chemical proxies to track the global movement of microplastics and tire wear particles. Understanding the transport kinetics of BTHs is essential for assessing the global footprint of non-exhaust emissions. Furthermore, BTHs are primarily investigated as markers of tire wear due to the massive scale of road traffic emissions; it is important to acknowledge that they are also present in other vulcanized rubber products (e.g., footwear, conveyor belts, and building materials). In indoor environments, these non-traffic sources may contribute to the observed concentrations. It is also worth noting that tire formulations are not static; industry practices and regulatory restrictions on specific vulcanization accelerators may have evolved over the 25-year period covered by this review. Such changes could potentially shift the chemical fingerprint of BTHs in the environment.

## Conclusions and final remarks

BTHs have been extensively studied in various environmental matrices, with a predominant focus on wastewater, soil, and leachates due to their strong association with urban sources, particularly tire wear particles. Analytical advancements have led to the widespread use of UHPLC-MS/MS for their detection, with methodologies evolving from traditional SPE extractions to innovative approaches like LVSPE and ml-SPE. Despite their confirmed environmental presence, studies on BTHs in particulate matter and non-exhaust vehicle emissions remain relatively limited.

To comprehensively evaluate the role of BTHs as tracers for TWP and non-exhaust emissions, it is essential to consider their distinct environmental behavior. Due to the possible volatilization followed by condensation or adsorption, as well as leaching processes, BTHs do not behave in the same way as the tire wear particles. While BTHs can improve our understanding of tire wear emissions and their environmental fate, they seem to primarily serve as indicators for other leachable compounds within tire wear particles. Additionally, the variability in the composition of benzothiazole species in tires should be acknowledged, as differences in their distribution can significantly impact their identification and quantification. Given that BTHs do not remain strongly bound within the TWP matrix, their quantification as markers presents notable challenges.

To enhance consistency and comparability across studies, the nomenclature of BTHs should be standardized in all publications. Future research should also focus on the broadest possible range of BTHs to deeply evaluate their reliability as non-exhaust tracers, especially considering that some studies suggest potential biogenic sources. If this characteristic is confirmed, particularly using aerosol samples, BTHs could be integrated into traffic emission models to improve our understanding of traffic-related pollution and its environmental impact.

Integrating the study of BTHs into wider debates on the circular economy and resource governance is essential. The data needed to accurately trace tire wear emissions can support the development of circular models, informing both the “eco-design” of tires with lower environmental footprints and the sustainable management of end-of-life tire waste streams. Furthermore, the push for advanced detection methods reviewed in this work, such as UHPLC-MS/MS, encourages innovation in analytical chemistry.

## Data Availability

The datasets used and/or analyzed during the current study are available from the corresponding author on reasonable request.

## References

[CR1] Akrochem (2023) RFQ. Physics and Technology of Linear Accelerator Systems. 10.1142/9789812703064

[CR2] Amey J, Mikkelsen Ø (2025) Fate of trace elements and emerging environmental pollutants (benzotriazoles and benzothiazoles) from a glacier-fed river in the mixing zone of an Arctic fjord system. Environ Chem Ecotoxicol 7:339–350. 10.1016/j.enceco.2025.01.001

[CR3] Asheim J, Vike-Jonas K, Gonzalez SV, Lierhagen S, Venkatraman V, Veivåg ILS, Snilsberg B, Flaten TP, Asimakopoulos AG (2019) Benzotriazoles, benzothiazoles and trace elements in an urban road setting in Trondheim, Norway: re-visiting the chemical markers of traffic pollution. Sci Total Environ 649:703–711. 10.1016/j.scitotenv.2018.08.299 30176481 10.1016/j.scitotenv.2018.08.299

[CR4] Asimakopoulos AG, Ajibola A, Kannan K, Thomaidis NS (2013a) Occurrence and removal efficiencies of benzotriazoles and benzothiazoles in a wastewater treatment plant in Greece. Sci Total Environ 452–453:163–171. 10.1016/j.scitotenv.2013.02.041 10.1016/j.scitotenv.2013.02.041 23500410

[CR5] Asimakopoulos AG, Wang L, Thomaidis NS, Kannan K (2013b) Benzotriazoles and benzothiazoles in human urine from several countries: a perspective on occurrence, biotransformation, and human exposure. Environ Int 59:274–281. 10.1016/j.envint.2013.06.007 23850588 10.1016/j.envint.2013.06.007

[CR6] Barbaro E, Feltracco M, Ulgelmo B, Spagnesi A, Frassati S, Mazzi G, Spolaor A, Barbante C, Gambaro A (2024) First evidence of benzothiazoles in arctic aerosols: seasonal trend and sources attribution. Sci Total Environ. 10.1016/j.scitotenv.2024.177722 10.1016/j.scitotenv.2024.177722 39581448

[CR7] Brownlee BG, Carey JH, MacInnis GA, Pellizzari IT (1992) Aquatic environmental chemistry of 2-(thiocyanomethylthio)benzothiazole and related benzothiazoles. Environ Toxicol Chem 11:1153–1168. 10.1002/etc.5620110812

[CR8] Campbell RH, Wise RW (1964a) Vulcanization. Part I. Fate of curing system during the sulfur vulcanization of natural rubber accelerated by benzothiazole derivatives. Rubber Chem Technol 37:635–649. 10.5254/1.3540356

[CR9] Campbell RH, Wise RW (1964b) Vulcanization. Part II. Fate of curing system during sulfur curing of Nr accelerated by MBT derivatives and activated by zinc stearate. Rubber Chem Technol 37:650–667. 10.5254/1.3540357

[CR10] Capolupo M, Sørensen L, Jayasena KDR, Booth AM, Fabbri E (2020) Chemical composition and ecotoxicity of plastic and car tire rubber leachates to aquatic organisms. Water Res. 10.1016/j.watres.2019.115270 10.1016/j.watres.2019.115270 31731243

[CR11] Cesaroni G, Boogaard H, Jonkers S, Porta D, Badaloni C, Cattani G, Forastiere F, Hoek G (2012) Health benefits of traffic-related air pollution reduction in different socioeconomic groups: the effect of low-emission zoning in Rome. Occup Environ Med 69:133–139. 10.1136/oem.2010.063750 21821870 10.1136/oem.2010.063750

[CR12] Chander Sharma P, Sharma D, Sharma A, Bansal KK, Rajak H, Sharma S, Thakur VK (2020) New horizons in benzothiazole scaffold for cancer therapy: advances in bioactivity, functionality, and chemistry. Appl Mater Today. 10.1016/j.apmt.2020.100783

[CR13] de Mendonça Ochs S, Souza TM, Sobrinho RDL, de Oliveira RB, Bernardes MC, Netto ADP (2023) Simultaneous evaluation of benzotriazoles, benzothiazoles and benzenesulfonamides in water samples from the impacted urban Jacarepaguá Lagoon System (Rio de Janeiro, Brazil) by liquid chromatography coupled to electrospray tandem mass spectrometry. Sci Total Environ. 10.1016/j.scitotenv.2022.160033 10.1016/j.scitotenv.2022.160033 36356777

[CR14] De Wever H, Besse P, Verachtert H (2001) Microbial transformations of 2-substituted benzothiazoles. Appl Microbiol Biotechnol 57:620–625. 10.1007/s00253-001-0842-2 11778869 10.1007/s00253-001-0842-2

[CR15] Deng C, Huang J, Qi Y, Chen D, Huang W (2022) Distribution patterns of rubber tire-related chemicals with particle size in road and indoor parking lot dust. Sci Total Environ 844:157144. 10.1016/j.scitotenv.2022.157144 35798097 10.1016/j.scitotenv.2022.157144

[CR16] Evans KS, Baqer D, Mafina M-K, Al-Sid-Cheikh M (2025) Qualitative and quantitative analysis of tire wear particles (TWPs) in road dust using a novel mode of operation of TGA-GC/MS. Environ Sci Technol Lett 12:79–84. 10.1021/acs.estlett.4c00937 39830726 10.1021/acs.estlett.4c00937 PMC11736838

[CR17] Favaro E, Mazzi G, Barbaro E, Masiol M, Alterio A, Gambaro A, Feltracco M (2024) Occurrence of tyre-derived particles in size-segregated aerosol in the urban area of Venice. Atmos Environ. 10.1016/j.atmosenv.2024.120784

[CR18] Favaro E, Barbaro E, Diémoz H, Gabrieli J, De Blasi F, Bortolini M, Munteanu A, Cozzi G, Cairns WRL, Barbante C, Gambaro A, Feltracco M (2026) Not so pristine: airborne benzothiazoles and organophosphate flame retardants in an alpine site under anthropogenic stress. Environ Pollut 390:127522. 10.1016/j.envpol.2025.127522 41389865 10.1016/j.envpol.2025.127522

[CR19] Feltracco M, Mazzi G, Barbaro E, Rosso B, Sambo F, Biondi S, Barbante C, Gambaro A (2023) Occurrence and phase distribution of benzothiazoles in untreated highway stormwater runoff and road dust. Environ Sci Pollut Res. 10.1007/s11356-023-30019-4 10.1007/s11356-023-30019-4 37740162

[CR20] Feltracco M, Mazzi G, Barbaro E, Gregoris E, Bortolini M, Barbante C, Gambaro A (2024) Insights into size-segregated distribution of benzothiazoles in indoor aerosol from office environments. Environ Sci Atmos 4:571–577. 10.1039/d4ea00031e

[CR21] Franklin EB, Alves MR, Moore AN, Kilgour DB, Novak GA, Mayer K, Sauer JS, Weber RJ, Dang D, Winter M, Lee C, Cappa CD, Bertram TH, Prather KA, Grassian VH, Goldstein AH (2021) Atmospheric benzothiazoles in a coastal marine environment. Environ Sci Technol 55:15705–15714. 10.1021/acs.est.1c04422 34787411 10.1021/acs.est.1c04422

[CR22] Fries E (2011) Determination of benzothiazole in untreated wastewater using polar-phase stir bar sorptive extraction and gas chromatography-mass spectrometry. Anal Chim Acta 689:65–68. 10.1016/j.aca.2011.01.015 21338758 10.1016/j.aca.2011.01.015

[CR23] Fuchte HE, Beck N, Bieg E, Bayer VJ, Achten C, Krauss M, Schäffer A, Smith KEC (2022) A look down the drain: identification of dissolved and particle bound organic pollutants in urban runoff waters and sediments. Environ Pollut 302:119047. 10.1016/j.envpol.2022.119047 35227846 10.1016/j.envpol.2022.119047

[CR24] Fussell JC, Franklin M, Green DC, Gustafsson M, Harrison RM, Hicks W, Kelly FJ, Kishta F, Miller MR, Mudway IS, Oroumiyeh F, Selley L, Wang M, Zhu Y (2022) A review of road traffic-derived non-exhaust particles: emissions, physicochemical characteristics, health risks, and mitigation measures. Environ Sci Technol 56:6813–6835. 10.1021/acs.est.2c01072 35612468 10.1021/acs.est.2c01072 PMC9178796

[CR25] Ginsberg G, Toal B, Kurland T (2011) Benzothiazole toxicity assessment in support of synthetic turf field human health risk assessment. J Toxicol Environ Health A 74:1175–1183. 10.1080/15287394.2011.586943 21797770 10.1080/15287394.2011.586943

[CR26] Grung M, Meland S, Ruus A, Ranneklev S, Fjeld E, Kringstad A, Rundberget JT, Dela Cruz M, Christensen JH (2021) Occurrence and trophic transport of organic compounds in sedimentation ponds for road runoff. Sci Total Environ. 10.1016/j.scitotenv.2020.141808 10.1016/j.scitotenv.2020.141808 32882565

[CR27] Gutiérrez-Martín D, Gil-Solsona R, Saaltink MW, Rodellas V, López-Serna R, Folch A, Carrera J, Gago-Ferrero P (2023) Chemicals of emerging concern in coastal aquifers: assessment along the land-ocean interface. J Hazard Mater. 10.1016/j.jhazmat.2023.130876 10.1016/j.jhazmat.2023.130876 36736215

[CR28] Halsband C, Sørensen L, Booth AM, Herzke D (2020) Car tire crumb rubber: does leaching produce a toxic chemical cocktail in coastal marine systems? Front Environ Sci. 10.3389/fenvs.2020.00125

[CR29] Jahanzab A, Zhao H, Lu R, Xie H-B (2025) Atmospheric oxidation mechanism of 2-hydroxy-benzothiazole initiated by hydroxyl radicals. ACS Earth Space Chem 9:457–466. 10.1021/acsearthspacechem.4c00230

[CR30] Jeong H, Li Z-M, Moon H-B, Kannan K (2026) Spatiotemporal distribution of 1,3-diphenylguanidine, benzotriazole, benzothiazole, N-(1,3-dimethylbutyl)-N′-phenyl-p-phenylenediamine, and their derivatives in surface and core sediments from Lake Sihwa. Korea. Environ Chem Ecotox 8:1466–1475. 10.1016/j.enceco.2026.03.011

[CR31] Jungclaus GA, Games LM, Hites RA (1976) Identification of trace organic compounds in tire manufacturing plant waste waters. Anal Chem 48:1894–1896. 10.1021/ac50007a021 970644 10.1021/ac50007a021

[CR32] Karimova NV, Wang W, Gerber RB, Finlayson-Pitts BJ (2024) Experimental and theoretical investigation of benzothiazole oxidation by OH in air and the role of O2. Environ Sci Process Impacts 26:2177–2188. 10.1039/D4EM00461B 39446060 10.1039/d4em00461b

[CR33] Kilpinen K, Devers J, Castro M, Tisler S, Jørgensen MB, Mortensen P, Christensen JH (2023) Catchment area, fate, and environmental risks investigation of micropollutants in Danish wastewater. Environ Sci Pollut Res Int 30:121107–121123. 10.1007/s11356-023-30331-z 37950122 10.1007/s11356-023-30331-z PMC10698095

[CR34] Kim L, Kim D, Kim SA, Kim H, Lee TY, An YJ (2022) Are your shoes safe for the environment? – toxicity screening of leachates from microplastic fragments of shoe soles using freshwater organisms. J Hazard Mater. 10.1016/j.jhazmat.2021.126779 10.1016/j.jhazmat.2021.126779 34352528

[CR35] Kim L, Lee TY, Kim H, An YJ (2022) Toxicity assessment of tire particles released from personal mobilities (bicycles, cars, and electric scooters) on soil organisms. J Hazard Mater. 10.1016/j.jhazmat.2022.129362 10.1016/j.jhazmat.2022.129362 35716575

[CR36] Kim L, Kim H, Lee TY, An YJ (2023) Chemical toxicity screening of tire particle leachates from vehicles and their effects on organisms across three trophic levels. Mar Pollut Bull. 10.1016/j.marpolbul.2023.114999 10.1016/j.marpolbul.2023.114999 37182239

[CR37] Klöckner P, Seiwert B, Eisentraut P, Braun U, Reemtsma T, Wagner S (2020) Characterization of tire and road wear particles from road runoff indicates highly dynamic particle properties. Water Res. 10.1016/j.watres.2020.116262 10.1016/j.watres.2020.116262 32798890

[CR38] Kloepfer A, Jekel M, Reemtsma T (2005) Occurrence, sources, and fate of benzothiazoles in municipal wastewater treatment plants. Environ Sci Technol 39:3792–3798. 10.1021/es048141e 15952387 10.1021/es048141e

[CR39] Kuntz V, Zahn D, Reemtsma T (2024) Quantification and occurrence of 39 tire-related chemicals in urban and rural aerosol from Saxony. Germany Environ Int 194:109189. 10.1016/j.envint.2024.109189 39671825 10.1016/j.envint.2024.109189

[CR40] Li Y-J, Ding W-H (2021) Determination of benzotriazole and benzothiazole derivatives in human urine by eco-friendly deep eutectic solvent-based ultrasound-assisted liquid-liquid microextraction followed by ultrahigh performance liquid chromatography quadrupole-time-of-flight mass spectrometry. Environ Pollut 284:117530. 10.1016/j.envpol.2021.117530 34261225 10.1016/j.envpol.2021.117530

[CR41] Li Z-M, Kannan K (2024) Mass loading, removal, and emission of 1,3-diphenylguanidine, benzotriazole, benzothiazole, N-(1,3-dimethylbutyl)-N′-phenyl-p-phenylenediamine, and their derivatives in a wastewater treatment plant in New York State, USA. ACS ES T Water 4:2721–2730. 10.1021/acsestwater.4c00221

[CR42] Li X, Wang L, Asimakopoulos AG, Sun H, Zhao Z, Zhang J, Zhang L, Wang Q (2018) Benzotriazoles and benzothiazoles in paired maternal urine and amniotic fluid samples from Tianjin, China. Chemosphere 199:524–530. 10.1016/j.chemosphere.2018.02.076 29455122 10.1016/j.chemosphere.2018.02.076

[CR43] Li ZM, Pal VK, Kannan P, Li W, Kannan K (2023) 1,3-Diphenylguanidine, benzothiazole, benzotriazole, and their derivatives in soils collected from northeastern United States. Sci Total Environ. 10.1016/j.scitotenv.2023.164110 10.1016/j.scitotenv.2023.164110 PMC10330497 37178851

[CR44] Li Y, Lu Z, Zhang X, Wang J, Zhao S, Dai Y (2024) Non-targeted analysis based on quantitative prediction and toxicity assessment for emerging contaminants in tire particle leachates. Environ Res. 10.1016/j.envres.2023.117806 10.1016/j.envres.2023.117806 38043899

[CR45] Liao C, Kim UJ, Kannan K (2018) A review of environmental occurrence, fate, exposure, and toxicity of benzothiazoles. Environ Sci Technol 52:5007–5026. 10.1021/acs.est.7b05493 29578695 10.1021/acs.est.7b05493

[CR46] Liu Y, Chen H, Li Y, Gao J, Dave K, Chen J, Li T, Tu R (2022) Exhaust and non-exhaust emissions from conventional and electric vehicles: a comparison of monetary impact values. J Clean Prod 331:129965. 10.1016/j.jclepro.2021.129965

[CR47] MacKenzie MJ, Hunter JV (1979) Sources and fates of aromatic compounds in urban stormwater runoff. Environ Sci Technol 13:179–183. 10.1021/es60150a011

[CR48] Mao W, Qu J, Liu H, Guo R, Liao K, Wu S, Hangbiao J, Hu Z (2024) Associations between urinary concentrations of benzothiazole, benzotriazole, and their derivatives and lung cancer: a nested case-control study. Environ Res 251:118750. 10.1016/j.envres.2024.118750 38522739 10.1016/j.envres.2024.118750

[CR49] Matamoros V, Jover E, Bayona JM (2010) Occurrence and fate of benzothiazoles and benzotriazoles in constructed wetlands. Water Sci Technol 61:191–198. 10.2166/wst.2010.797 20057105 10.2166/wst.2010.797

[CR50] Maurer L, Carmona E, Machate O, Schulze T, Krauss M, Brack W (2023) Contamination pattern and risk assessment of polar compounds in snow melt: an integrative proxy of road runoffs. Environ Sci Technol 57:4143–4152. 10.1021/acs.est.2c05784 36862848 10.1021/acs.est.2c05784 PMC10018729

[CR51] Mazzi G, Feltracco M, Barbaro E, Scalabrin E, Favaro E, Colombi C, Liu G, Yang Y, Gambaro A (2026) Tracking the source: first evidence of benzothiazoles in outdoor airport aerosol. Environ Chem Ecotoxicol 8:66–75. 10.1016/j.enceco.2025.11.011

[CR52] Möhlau R, Krohn CW (1888) Ueber die Umwandlungen des Dimethylanilins und Monomethylanilins unter dem Einfluss des Schwefels. Ber Dtsch Chem Ges 21:59–67. 10.1002/cber.18880210116

[CR53] Mol Z, Walgraeve C, De Pril R, Van Langenhove H, Demeestere K (2024) Trace analysis of taste and odour compounds in drinking water by stir bar sorptive extraction followed by thermal desorption - gas chromatography - mass spectrometry (SBSE-TD-GC-MS). Sci Total Environ. 10.1016/j.scitotenv.2024.176878 10.1016/j.scitotenv.2024.176878 39423895

[CR54] Ng K, Alygizakis N, Nika MC, Galani A, Oswald P, Oswaldova M, Čirka Ľ, Kunkel U, Macherius A, Sengl M, Mariani G, Tavazzi S, Skejo H, Gawlik BM, Thomaidis NS, Slobodnik J (2023) Wide-scope target screening characterization of legacy and emerging contaminants in the Danube River Basin by liquid and gas chromatography coupled with high-resolution mass spectrometry. Water Res. 10.1016/j.watres.2022.119539 10.1016/j.watres.2022.119539 36610182

[CR55] Ni HG, Lu FH, Luo XL, Tian HY, Zeng EY (2008) Occurrence, phase distribution, and mass loadings of benzothiazoles in riverine runoff of the Pearl River Delta. China Environ Sci Technol 42:1892–1897. 10.1021/es071871c 18409609 10.1021/es071871c

[CR56] Nuñez A, Vallecillos L, Marcé RM, Borrull F (2020) Occurrence and risk assessment of benzothiazole, benzotriazole and benzenesulfonamide derivatives in airborne particulate matter from an industrial area in Spain. Sci Total Environ 708:135065. 10.1016/j.scitotenv.2019.135065 31787291 10.1016/j.scitotenv.2019.135065

[CR57] Núñez M, Fontanals N, Borrull F, Marcé RM (2022) Multiresidue analytical method for high production volume chemicals in dust samples, occurrence and human exposure assessment. Chemosphere. 10.1016/j.chemosphere.2022.134639 10.1016/j.chemosphere.2022.134639 35447216

[CR58] Pillard DA, Cornell JS, DuFresne DL, Hernandez MT (2001) Toxicity of benzotriazole and benzotriazole derivatives to three aquatic species. Water Res 35:557–560. 10.1016/S0043-1354(00)00268-2 11229011 10.1016/s0043-1354(00)00268-2

[CR59] Que DE, Wang X, Nilsson S, Zammit I, Muir DCG, Rauert C, Toms L-M, Prasad P, Shiels RG, Eaglesham G, Hobson P, Langguth D, Mueller JF (2024) Trends of benzotriazoles and benzothiazoles in Australian pooled urine samples from 2012 to 2023. Environ Sci Technol 58:19960–19969. 10.1021/acs.est.4c08824 39475159 10.1021/acs.est.4c08824

[CR60] Reemtsma T, Weiss S, Mueller J, Petrovic M, González S, Barcelo D, Ventura F, Knepper TP (2006) Polar pollutants entry into the water cycle by municipal wastewater: a European perspective. Environ Sci Technol 40:5451–5458. 10.1021/es060908a 16999124 10.1021/es060908a

[CR61] Ren Y, Li W, Jia Q, Zhao Y, Qu C, Liu L, Liu J, Wu C (2024) Separation and quantification of tire and road wear particles in road dust samples: bonded-sulfur as a novel marker. J Hazard Mater. 10.1016/j.jhazmat.2023.133089 10.1016/j.jhazmat.2023.133089 38016316

[CR62] Salas D, Borrull F, Marcé RM, Fontanals N (2016) Study of the retention of benzotriazoles, benzothiazoles and benzenesulfonamides in mixed-mode solid-phase extraction in environmental samples. J Chromatogr A 1444:21–31. 10.1016/j.chroma.2016.03.053 27040512 10.1016/j.chroma.2016.03.053

[CR63] Spies RB, Andresen BD, Rice DW Jr (1987) Benzthiazoles in estuarine sediments as indicators of street runoff. Nature 327:697–699. 10.1038/327697a0

[CR64] Stasinakis AS, Thomaidis NS, Arvaniti OS, Asimakopoulos AG, Samaras VG, Ajibola A, Mamais D, Lekkas TD (2013) Contribution of primary and secondary treatment on the removal of benzothiazoles, benzotriazoles, endocrine disruptors, pharmaceuticals and perfluorinated compounds in a sewage treatment plant. Sci Total Environ 463–464:1067–1075. 10.1016/j.scitotenv.2013.06.087 10.1016/j.scitotenv.2013.06.087 23891999

[CR65] Tan J, Shen A, Guo Y, Wu J, Shen H, Zhang T (2026) Sources, hazards, and formation mechanisms of organic emissions from crumb rubber modified asphalt: a review. J Environ Chem Eng 14:121444. 10.1016/j.jece.2026.121444

[CR66] Thomas J, Moosavian SK, Cutright T, Pugh C, Soucek MD (2022) Method development for separation and analysis of tire and road wear particles from roadside soil samples. Environ Sci Technol 56:11910–11921. 10.1021/acs.est.2c03695 35980850 10.1021/acs.est.2c03695

[CR67] Wang W, Park S, Choi BG, Oh JE (2023) Corrigendum to “occurrence and removal of benzotriazole and benzothiazole in drinking water treatment plants” [Environ Pollut 316 (2023) 120563](S0269749122017778). Environ Pollut. 10.1016/j.envpol.2022.120563 10.1016/j.envpol.2022.120563 36332710

[CR68] Wei L-N, Wu N-N, Xu R, Liu S, Li H-X, Lin L, Hou R, Xu X-R, Zhao J-L, Ying G-G (2024) First evidence of the bioaccumulation and trophic transfer of tire additives and their transformation products in an estuarine food web. Environ Sci Technol 58:6370–6380. 10.1021/acs.est.3c10248 38497719 10.1021/acs.est.3c10248

[CR69] Wik A, Dave G (2009) Occurrence and effects of tire wear particles in the environment - a critical review and an initial risk assessment. Environ Pollut 157:1–11. 10.1016/j.envpol.2008.09.028 18990476 10.1016/j.envpol.2008.09.028

[CR70] Wu X, Zhu Y, Guo R, Huang J, Jin H, Zhou L (2024) 2-Mercaptobenzothiazole-derived vulcanization accelerators in urine samples from Chinese adults. Sci Total Environ. 10.1016/j.scitotenv.2024.176815 10.1016/j.scitotenv.2024.176815 39393704

[CR71] Xu W, Zhang L, Tian Y, Zhu X, Han X, Miao L, Yan W (2022) Occurrence and distribution of organic corrosion inhibitors (OCIs) in riverine sediments from the Pearl River Delta, South China. Environ Sci Pollut Res 29:76961–76969. 10.1007/s11356-022-21192-z 10.1007/s11356-022-21192-z 35670946

[CR72] Xue J, Lin Y, Zhao D, Kannan K (2024) Occurrence, removal, and fate of benzothiazoles (BTHs) and benzotriazoles (BTRs) in two wastewater treatment plants in New York State, USA. Sci Total Environ. 10.1016/j.scitotenv.2024.175090 10.1016/j.scitotenv.2024.175090 39079646

[CR73] Yin X, Wang L, Mao L (2024) Comparing the developmental toxicity delay and neurotoxicity of benzothiazole and its derivatives (BTHs) in juvenile zebrafish. Toxics 12:341. 10.3390/toxics12050341 38787120 10.3390/toxics12050341 PMC11125584

[CR74] Zhan Y, Huang DD, Wang H, Gao Y, Li Y, Zhu S, Liu Q, Duan J, Yang L, Xu W, Zhong H, Zhou L, Li YJ, Huang C, Fu Q, Hoffmann T, Huang R-J (2025) Formation kinetics and yields of secondary organic aerosol from benzothiazoles based on oxidation flow reactor and ambient studies. Environ Sci Technol Lett 12:1366–1372. 10.1021/acs.estlett.5c00714

[CR75] Zhang J, Zhang X, Wu L, Wang T, Zhao J, Zhang Y, Men Z, Mao H (2018) Occurrence of benzothiazole and its derivates in tire wear, road dust, and roadside soil. Chemosphere 201:310–317. 10.1016/j.chemosphere.2018.03.007 29525659 10.1016/j.chemosphere.2018.03.007

[CR76] Zhang H-Y, Huang Z, Liu Y-H, Hu L-X, He L-Y, Liu Y-S, Zhao J-L, Ying G-G (2023a) Occurrence and risks of 23 tire additives and their transformation products in an urban water system. Environ Int 171:107715. 10.1016/j.envint.2022.107715 36577297 10.1016/j.envint.2022.107715

[CR77] Zhang R, Zhao S, Liu X, Tian L, Mo Y, Yi X, Liu S, Liu J, Li J, Zhang G (2023b) Aquatic environmental fates and risks of benzotriazoles, benzothiazoles, and *p*-phenylenediamines in a catchment providing water to a megacity of China. Environ Res 216:114721. 10.1016/j.envres.2022.114721 36343716 10.1016/j.envres.2022.114721

[CR78] Zhao ML, Fu J, Ji X, Zhang J, He Z, Yang GP (2024) Comprehensive analysis of benzothiazoles (BTHs), benzotriazoles (BTRs), and benzotriazole ultraviolet absorbers (BUVs) in the western South China Sea: spatial distributions, migration tendencies and ecotoxicological relevance. Water Res. 10.1016/j.watres.2024.122372 10.1016/j.watres.2024.122372 39241383

[CR79] Zhao ML, Ji X, He Z, Yang GP (2024) Spatial distribution, partitioning, and ecological risk assessment of benzotriazoles, benzothiazoles, and benzotriazole UV absorbers in the eastern shelf seas of China. Water Res. 10.1016/j.watres.2023.120885 10.1016/j.watres.2023.120885 38016257

[CR80] Zhao ML, Ji X, Zhang J, Yang GP (2024) Spatiotemporal variation, partitioning, and ecological risk assessment of benzothiazoles, benzotriazoles, and benzotriazole UV absorbers in the Yangtze River Estuary and its adjacent area. J Hazard Mater. 10.1016/j.jhazmat.2023.133337 10.1016/j.jhazmat.2023.133337 38142656

